# Precise genome modification in tomato using an improved prime editing system

**DOI:** 10.1111/pbi.13497

**Published:** 2020-11-25

**Authors:** Yuming Lu, Yifu Tian, Rundong Shen, Qi Yao, Dating Zhong, Xuening Zhang, Jian‐Kang Zhu

**Affiliations:** ^1^ Shanghai Center for Plant Stress Biology CAS Center for Excellence in Molecular Plant Sciences Chinese Academy of Sciences Shanghai China; ^2^ Department of Horticulture and Landscape Architecture Purdue University West Lafayette IN USA

**Keywords:** prime editing, genome editing, tomato, CRISPR, Cas9

The CRISPR/Cas‐mediated genome editing technology has been widely applied to create knockout alleles of genes by generating short insertions or deletions (indel) in various plant species. Due to the low efficiency of homology‐directed repair (HDR) and difficulties in the delivery of DNA template for HDR, precise genome editing remains challenging in plants (Mao *et al*., 2019). A tandem repeat‐HDR method was developed very recently for sequence replacement in rice, which is most useful for monocots (Lu *et al*., 2020). Base editors developed from Cas9 nickase fusion with cytosine and adenine deaminases enable targeted C‐to‐T or A‐to‐G substitutions, but are restricted to specific types of base replacements and target site selections (Mao *et al*., 2019). A ‘search‐and‐replace’ method, also known as prime editing, was developed in mammalian cells, which enables user‐defined sequence changes on a target site without requiring DSBs or the delivery of DNA repair templates (Anzalone *et al*., 2019). Several research groups have adopted this method for use in monocotyledonous plants, including rice and wheat (Butt *et al*., 2020; Hua *et al*., 2020; Li *et al*., 2020; Lin *et al*., 2020; Tang *et al*., 2020; Xu *et al*., 2020). For reasons that are still unclear, although base editing has been highly efficient in monocots such as rice, its efficiencies are very low in dicots (Kang *et al*., 2018; Mao *et al*., 2019). Whether prime editing can be used for dicotyledonous plants such as tomato, is unknown. Here, we report successful adoption of prime editors for use in tomato through codon and promoter optimization.

The prime editing system consists of three parts: an nCas9‐MMLV (engineered Moloney murine leukaemia virus reverse transcriptase) fusion protein, a prime editing guide RNA (pegRNA) and a small guide RNA (sgRNA) for nicking. We incorporated the mammalian prime editing system into a plant binary vector for expression in tomato, generating pCXPE01. As shown in Figure 1a, the commonly used CaMV 35S promoter (2x35S) was used to express the nCas9‐hMMLV (human codon‐optimized MMLV) fusion protein while pegRNA and sgRNA were driven by the U6 promoter of *Arabidopsis*. In order to test whether the system may work in tomato, we constructed a dual‐luciferase reporter system, where the NanoLuc, an engineered super sensitive luciferase, was completely disabled by introducing frame‐shift mutations, a two nucleotide deletion and six nucleotide substitution (NanoLucM). Only precise editing on NanoLucM can restore its luciferase activity, and the efficiency could be sensitively quantified through luminescence measurement, using the firefly luciferase as an internal control (Figure 1b). Two pegRNAs, pegRNA‐12 and pegRNA‐13, were designed with 13 and 14 nt PBS (primer binding site), respectively, and a 23 nt RT (reverse transcription) template. Each was accompanied by a sgRNA for nicking at a site located 32‐nt or 49‐nt downstream from the pegRNA nicking sites (Figure 1b). The two pCXPE01 constructs were each introduced into tomato leaves together with the Dual‐LucM reporter using biolistic bombardment. Five days later, we detected the restored luminescence in both samples of pegRNA‐12 and pegRNA‐13, with an average efficiency of 0.26% compared with the control Dual‐Luc that was counted as 100%. These results indicated that the primer editor can be used in tomato.

**Figure 1:**
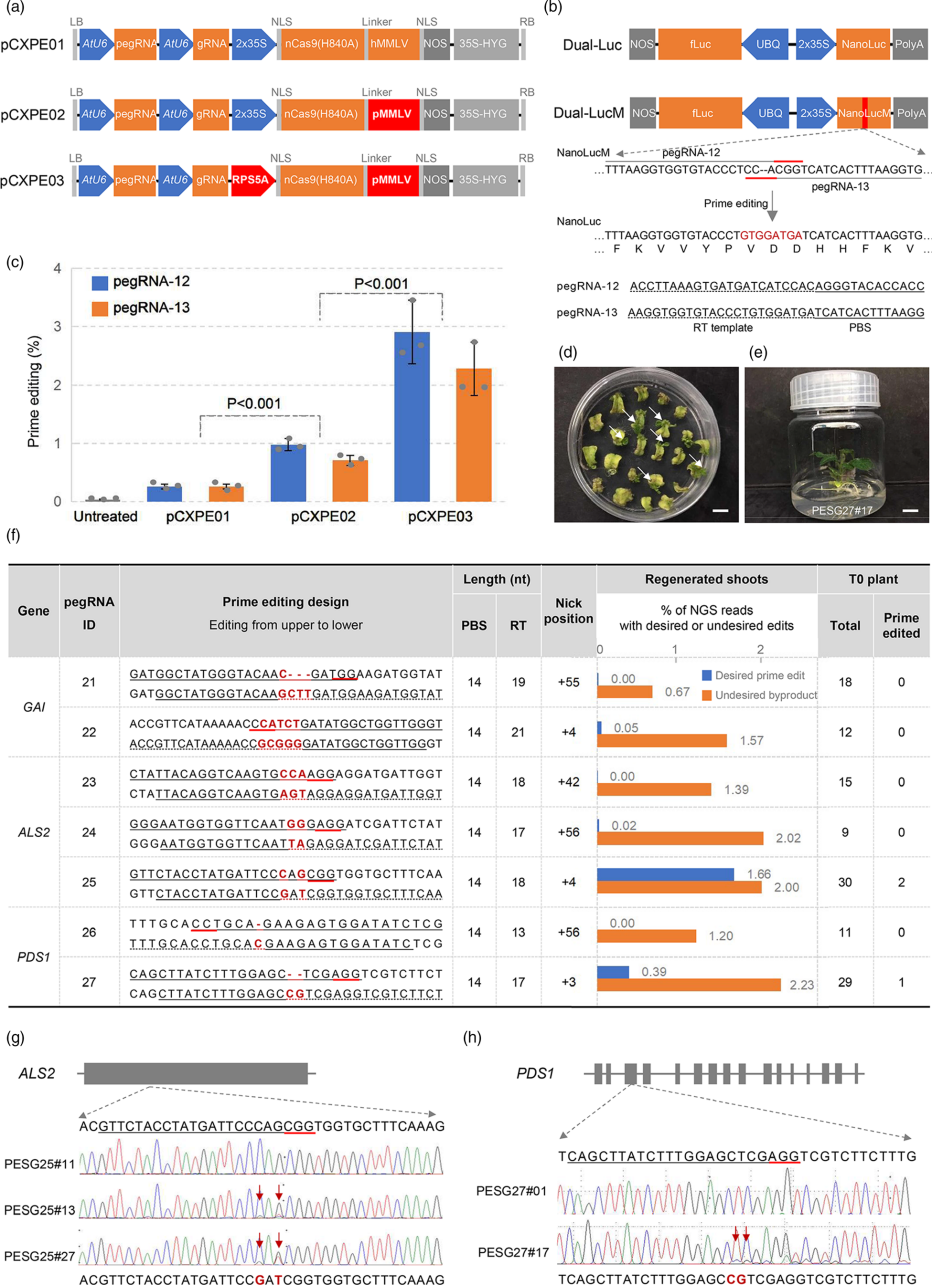
Prime editing for precise genome modification in tomato. (a) Schematic diagram of the prime editing constructs in this study. (b) Dual‐luciferase reporter system for assessments of prime editing efficiencies. Dual‐LucM contains an inactive NanoLuc designated as NanoLucM. pegRNA‐12 and pegRNA‐13 target the mutated site to restore the NanoLuc activity. fLuc, firefly luciferase. (c) Comparison of prime editing efficiencies of pCXPE01, pCXPE02 and pCXPE03 in tomato using the Dual‐luciferase reporter system delivered by bombardment. Editing frequencies were calculated by NanoLuc/fLuc, counting the normal reporter Dual‐Luc as 100%. Values (mean ± s.e.m.) were calculated from three independent experiments (*n* = 3). P values were obtained using the two‐tailed Student’s *t*‐test. (d and e) Regenerated tomato shoots (d, indicated by arrows) and a representative T0 seedling (e) on hygromycin‐containing medium. Bar, 10 mm. (f) Summary of prime editing results of pCXPE03 in regenerated tomato shoots and T0 plantlets, as determined by NGS and Sanger sequencing, respectively. (g and h) Sequence chromatograms of prime‐edited T0 plants. Edited bases were indicated by red arrows. (b, f, g and h) Targets and their PAMs in sequences were underlined in black and red, respectively. PBS and RT sequences are underlined with solid and dashed lines, respectively. Nucleotides for substitutions are marked in red.

Previous studies on base editing in dicots showed that improvement of nCas9 expression level could significantly increase the editing efficiency (Kang *et al*., 2018). Therefore, we sought to optimize pCXPE01 to improve editing efficiency by increasing nCas9‐MMLV expression level. We replaced the hMMLV with a plant codon‐optimized MMLV (pMMLV), generating pCXPE02. Transient expression assays on the pegRNA‐12 and pegRNA‐13 sites using the same Dual‐LucM reporter described above resulted in a 3.2‐fold improvement compared with that of pCXPE01 (0.85% vs. 0.26%). Then, we replaced the 35S promoter with the ribosomal protein S5A (*RPS5A*) promoter of tomato (pCXPE03), which increased the average efficiency to 2.6%, approximately 10 times higher than that of the original pCXPE01 (Figure 1c). Such improvements are consistent with previous reports of using the RPS5A promoter to improve base editing efficiencies in the dicotyledonous plant *Arabidopsis* (Kang *et al*., 2018). The improved prime editing frequency in tomato leaves was comparable to that in monocots reported recently.

To determine whether the optimized primer editor pCXPE03 may be used to edit endogenous genes in tomato, three tomato genes, *GAI* (Solyc11g011260), *ALS2* (Solyc03g044330) and *PDS1* (Solyc03g123760), were tested. In order to make a clear distinction between prime editing and the random indels caused by nCas9, multiplex base substitutions and/or insertions were designed for introduction into these genes using a total of seven pegRNAs (Figure 1f). Each pegRNA contains a 14 nt PBS and a 13–21 nt RT template. Corresponding plasmids were constructed using pCXPE03 and introduced into tomato (Micro‐Tom) using *Agrobacteria*. After six weeks of selection on hygromycin‐containing medium, 280 regenerated shoots (Figure 1d) were mixed together for DNA extraction and genotyping using next‐generation sequencing (NGS). Analysis of a total of 12,820,501 NGS reads detected desired prime editing sequences in four pegRNA sites, including pegRNA‐22, pegRNA‐24, pegRNA‐25 and pegRNA‐27, with frequencies ranging from 0.025% to 1.66% (Figure 1f). Such efficiencies were comparable with the reported prime editing results in rice determined using NGS. Similar to the prime editing in rice, undesired by‐product sequences were also observed at all targets in tomato at frequencies ranging from 0.5% to 4.9%, possibly due to the nicking activity of nCas9.

We regenerated hundreds of T0 tomato plants (Figure 1e) for the three genes and genotyped 124 transgenic seedlings using Sanger sequencing. According to the sequencing results, we detected desired edits at two genes, *ALS2* and *PDS1*. We found that 2 out of 30 ALS2 lines (PESG25#13 and #27, 6.7%) contained the desired CAG‐to‐GAT multi‐nucleotide substitutions at the pegRNA‐25 site, resulting in the S642I amino acid change in ALS2. For the *PDS1* that encodes the essential phytoene desaturase, we identified one mutant PESG27#17 (1 out of 29, 3.4%) having the designed CG‐insertion at the pegRNA‐27 site. In all previous reports of prime editing on endogenous genes in wheat and rice, only chimeric or heterozygous edited plants were produced except for one pegRNA targeting *OsALS2* that produced one homozygous rice line. Accordingly, no plant phenotype results were reported in these studies. Similarly, our prime‐edited T0 tomato plants were chimeras and did not display any obvious phenotypes (Figure 1e, g and h). Therefore, for both monocots and dicots, assessment of the utility of prime editing awaits future analysis of large populations of edited lines and their off‐springs. Editing frequencies vary at the seven sites. Higher efficiencies at the pegRNA‐22, pegRNA‐25 and pegRNA‐27 sites may be due to the nicking positions of sgRNA (+4, +4, +3) that were located closer to the pegRNAs, consistent with the PE3 design strategy of prime editing in mammalian cells. We note that multiplex base substitutions and/or insertions were tested here; it is possible prime editing may yield higher efficiencies for simpler base changes (e.g. one‐nucleotide substitution; Anzalone *et al*., 2019). Regardless, the editing results here suggest that we can use pCXPE03 for prime editing in tomato.

Compared to that in monocots, base editors do not function well in dicots and thus need to be improved (Kang *et al*., 2018). Here, through codon and promoter changes, we have improved the efficiency of prime editing considerably in tomato, to levels comparable to those in rice. Further improvements would make prime editing a useful tool for precise genome editing in plant research and breeding.

## Competing financial interests

The authors declare no competing financial interests.

## Author contributions

Y.L. and Y.T. designed the experiments; Y.L., Y.T., R.S., Q.Y., X.Z. and D.Z. performed the experiments; Y.L. and Y.T. wrote the manuscript. J.‐K.Z. supervised the project and edited the manuscript.
